# In vitro assessment of Cowpea cv. GIZA-18 forage grown from low-pressure radiofrequency plasma-treated seeds under salt stress

**DOI:** 10.1038/s41598-026-37598-5

**Published:** 2026-02-19

**Authors:** Mohamed H. Shokry, Hani S. Saudy, Gouda F. Gouda, Abeer M. EL-Essawy, Seham A. Hashem, Marwa A. Madcour, Nasr E. El-Bordeny, Abdelfattah T. Elgendy

**Affiliations:** 1 Department of Environmental Agricultural Science, Faculty of Graduate Studies and Environmental Research, Ain Shams University, Cairo, Egypt; 2 Agronomy Department, Faculty of Agriculture, Ain Shams University, Hadayek Shoubra, P.O. Box 68, Cairo, 11241 Egypt; 3 Animal Production Department, Faculty of Agriculture, Ain Shams University, Cairo, Egypt; 4 Department of Animal and Fish Production, College of Agricultural and Food Sciences, King Faisal University, Al-Ahsa, Saudi Arabia; 5 Animal and Poultry Nutrition Department, Animal and Poultry Division, Desert Research Center, Mataryia, Cairo, Egypt; 6 Animal Nutrition Department, Animal Production Research Institute, Agriculture Research Center, Dokki, Giza, Egypt; 7 Physics Department, Faculty of Science, Ain Shams University, Cairo, Egypt

**Keywords:** Anti-nutritional agents, Cowpea forage quality, *In vitro* degradability, Livestock, Plasma technology, Salinity stress, Seed priming, Physiology, Plant sciences

## Abstract

Soil salinization, as one of the most critical phenomena of climate change, poses significant challenges to agricultural productivity, especially in arid and semi-arid regions like Egypt’s Nile Delta surrounding area. Forage crops, such as cowpea (*Vigna unguiculata*), demonstrate relative tolerance under harsh conditions; however, its productivity and nutritional quality may be compromised by abiotic stress. Plasma as an advanced and precise technology showed promising results in agriculture. However, insights into the role of plasma in stimulating plant tolerance to salinity while maintaining high-quality products require in-depth investigations and studies. This study evaluated the effects of plasma application as a recent method for seed priming on forage productivity, anti-nutritional factors (ANFs), and in vitro degradability dynamics of cowpea cv. GIZA-18 under salinity stress. Seeds were exposed to low-pressure radiofrequency plasma (13.56 MHz, 60 W) for 0.0 (control), 1.0, 2.0, and 3.0 min (min) and seedling growth traits were evaluated under wire house conditions. Next, in two seasons of 2022 and 2023, plasma-treated seeds for 0.0, 1.0, and 2.0 min were cultivated under field conditions in soil with three levels of salinity expressed in electrical conductivity (EC) of 3.0, 5.5, and 7.0 dS m⁻¹. Findings exhibited that chlorophyll concentration, length, and weight of cowpea seedlings were improved by the application of different doses of plasma. Plasma dose of 1.0 min under moderate salinity (EC5.5) resulted in promising and established outputs, as the improvements in forage yield amounted to 47.0 and 146.1% in the first and second season, respectively. Results also demonstrated that based on the dry matter (DM) basis, plasma treatment significantly reduced ANFs, with 1.0 min exposure decreasing tannins by 15.72% (from 9.54 mg /100 g DM to 8.04 mg /100 g DM) and saponins by 48.39% (from 1.55 g/100 g DM to 0.80 g/100 g DM). At high salinity (EC7.0), crude protein increased to 166.9 g/kg in plants produced from 1.0 min treated seeds. Cowpea forage grown from plasma-treated seeds for 1.0 min showed significantly higher in vitro degradable dry matter (IVDDM), in vitro degradable neutral detergent fiber (IVDNDF), and in vitro degradable acid detergent fiber (IVDADF) compared to both untreated and 2.0-min treated seeds. Fermentation kinetics indicated that the 1.0-min of plasma treatment significantly reduced gas production per gram of DM and gas production per gram of organic matter (OM) in comparison to untreated seeds (0.0 min). Additionally, plants grown from seeds treated for 2-min had significantly higher ammonia concentrations compared to those grown from untreated seeds or those treated for 1.0 min. In brief, we can conclude that plasma technology can contribute to enhancing the establishment of cowpeas grown in saline soils, improving seedling growth, salinity tolerance, and productivity. Further, the In Vitro evaluation confirmed plasma’s efficacy in ameliorating the nutritional quality of cowpea forage under salt stress. Accordingly, cowpea growers are advised to subject the seed to plasma for 1.0 min immediately prior to sowing in saline soil for keeping forage productivity and quality.

## Introduction

Climate change poses a global threat with regionally specific consequences, disproportionately affecting developing countries with limited adaptive capacity^1^. As climate variability intensifies, global food security in such regions is under growing pressure, especially with population growth expected to reach 8.5 to 12 billion by 2100, with over 75% residing in developing nations across Africa and Asia^1^. Egypt as one of the developing countries is projected to suffer significant impacts from sea level rise, saltwater intrusion, and reduced agricultural productivity, all of which threaten live hoods and food security in the region^2^.

Agriculture is intrinsically linked to climate change, both as a contributor to greenhouse gas emissions and as a sector highly susceptible to environmental fluctuations. Elevated temperatures, altered precipitation patterns, and extreme weather events reduce crop yields and degrade soil health. While higher atmospheric CO₂ may enhance certain plant functions, the overall impact on productivity is predominantly negative^3^, especially in salt-prone regions^4,5^. As a result of a changing climate, the agro-activity and agronomic production face vehement ecological perturbations such as drought, disadvantageous temperatures, unhealthy soils, and salinization^6–11^. The dominance of these stresses in the environments surrounding plants certainly leads to dire consequences, harming plant functions and growth^12–17^. One of the most pressing challenges in Egyptian agriculture is soil salinity, which restricts crop performance and quality^18,19^. According to plant responses, salinity is one of the deleterious stressors for plant growth, as lifted salinity levels in soil can handicap plant development via physiological drought and ion toxicity^20–22^. Indirectly, production in livestock as a major agricultural sector is also deeply intertwined with climate change. Feed availability and quality are key influential factors in expanding ruminant and poultry production systems. As salinity impacts forage growth and nutritional content, feed costs rise, affecting the entire food supply chain^19,23^. Therefore, crops grown in saline soils must be protected through effective applications to ensure thriving growth, acceptable productivity and feed supply for livestock^24,25^.

To ensure sustainable crop production with high-quality and abundant forages, precise and sophisticated methods must be employed, taking environmental security into account^26,27^. Under low temperatures and pressures, plasmas can be formed to utilize advantage of the synergistic action of high-energy molecules, which include blustery atoms, reactive particles, and electromagnetic emissions^28,29^. The foundational plasma modeling efforts offer deep insights into sheath dynamics and plasma behavior in industrial systems, potentially informing large-scale agricultural applications of plasma-based technologies^30,31^. Several studies support the potential of plasma in agriculture. In this respect, the use of plasma technology to stimulate seed germination and enhance crop productivity could represent a promising and innovative approach, especially in facing environmental challenges such as soil salinity^32,33^. Recent studies have shown that plasma treatment of seeds can significantly improve germination and growth rates without altering the genetic structure of the plant, positioning plasma as a safe and effective epigenetic stimulant^34,35^. Plasma treatment improves water uptake and accelerates germination benefits, particularly critical in saline soils^36,37^. Also, Dufour et al.^38^ documented enhancements in seedling vigor, plant height, and early activity, all contributing to improved crop yields.

Cowpea is a suitable candidate for cultivation in arid areas due to its inherent tolerance to drought, as well as its nutritional value as a forage crop. As forage, cowpea provides high protein content ranging from 14% to 21% in fresh leaves and supplies essential nutrients for livestock, contributing significantly to feed quality during dry seasons^39^. Moreover, it has the ability to fix atmospheric nitrogen improving soil fertility and support sustainable cropping systems, especially when intercropped with cereals like maize or millet^40–42^. Cowpea is a drought-resilient legume widely cultivated in arid and semi-arid regions, making it well-suited for marginal lands with limited water availability. These attributes make cowpea an efficient dual-purpose crop that addresses both environmental stress and nutritional demand in livestock systems. However, cowpea can tolerate salinity levels not more than 4.8 dS m^− 1^ with moderate reductions in biomass yield^43^. On the other hand, the cultivation of forage crops in marginal lands becomes increasingly important, particularly when aided by efficient techniques like plasma treatment, which enhances seed performance under saline conditions^44^.

Accordingly, as a serious attempt, the current work hypothesized that plasma as precise applications could contribute to enhancing the tolerance of cowpea to salt stress to obtain high forage productivity with excellent quality. Therefore, this study aimed to investigate the use of plasma-treated cowpea (GIZA-18) seeds to improve crop yields in saline environments and enhance the nutritional value of the resulting forage for animal feed. Herein, the influences of plasma treatment at different doses on cowpea productivity and the in vitro digestibility of forage under varying salinity levels were assessed.

## Materials and methods

### Plasma execution

#### Plasma treatment setup

**Fig. 1:**
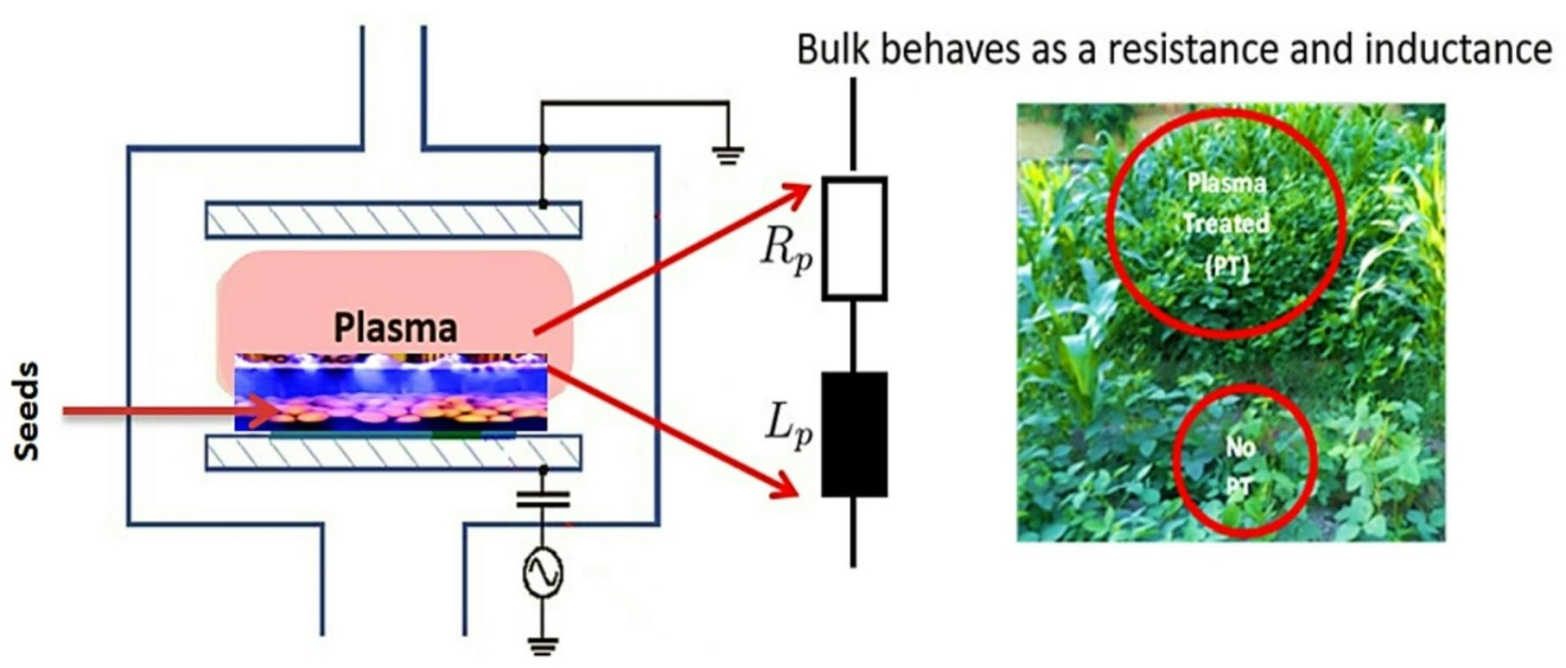
Schematic diagram of the low-pressure 13.56 MHz RF capacitive plasma reactor (PICO system) used in seed treatment. Operating power: 60 W.

Cowpea seeds (*Vigna unguiculata* cv. GIZA-18) were exposed to surface modification using a low-pressure, non-thermal radiofrequency (RF) plasma system operating at 13.56 MHz. The plasma reactor, illustrated schematically in Fig. 1, was a cylindrical plate capacitive discharge system (PICO plasma system) designed to receive a good number of seeds and ensure uniform plasma exposure. The seeds were placed at a specified distance from the plasma discharge zone and subjected to controlled rotation during treatment to ensure uniform surface exposure and minimize localized heating or thermal damage.

#### Plasma process parameters

**Fig. 2:**
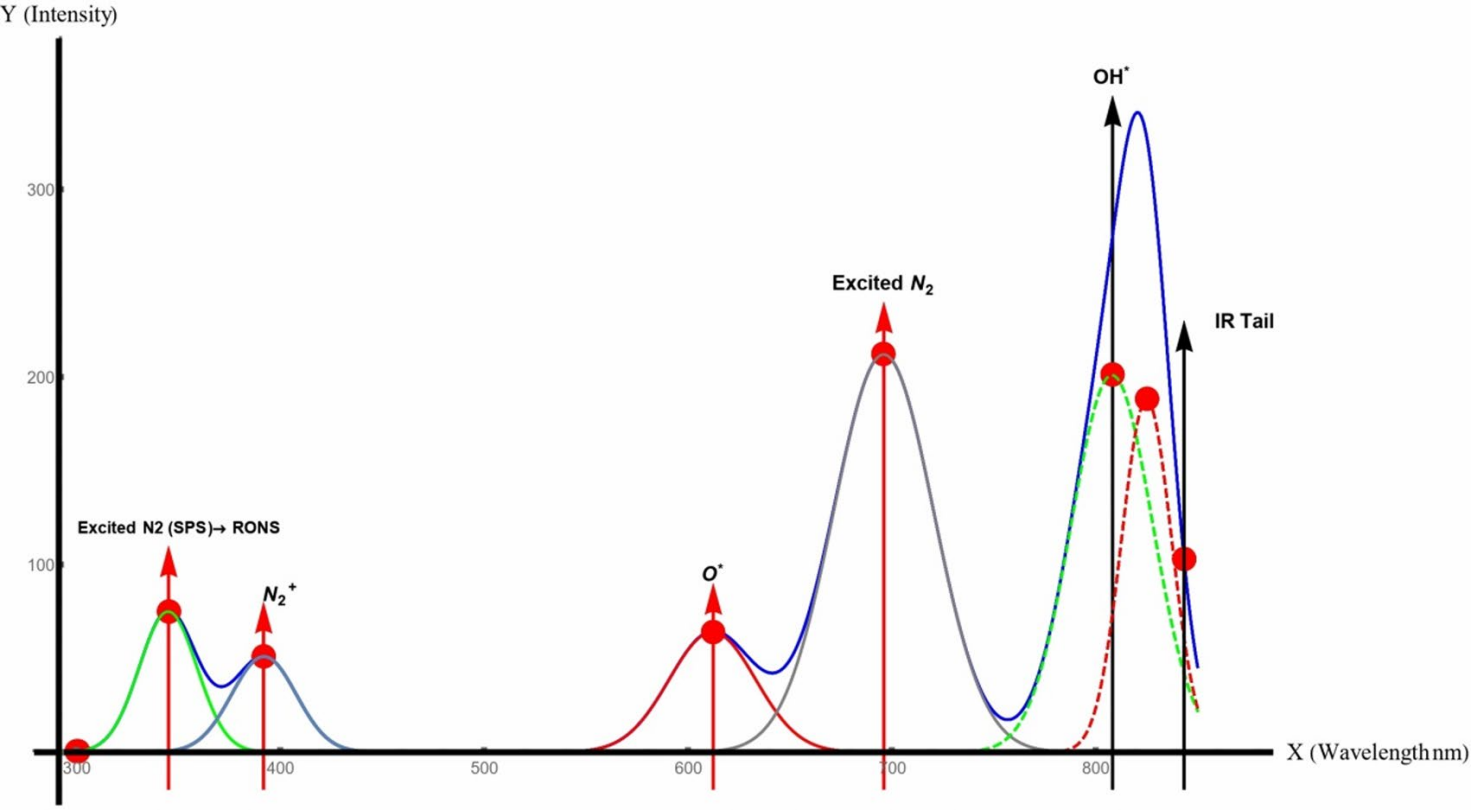
Emission spectrum of cold plasma used for the treatment of Giza-18 seeds. Arrows above each peak indicate the expected functional role of the corresponding plasma free radical species in generating reactive oxygen and nitrogen species (RNOS), including OH• radicals, excited oxygen (O*), and nitrogen (N) states. IR: energy tail contribution.

Parameters illustrated in Table 1 were selected based on previous literature and optimized to avoid thermal degradation while confirming sufficient interaction between plasma-generated reactive species, such as reactive oxygen and nitrogen species (RNS), and the seed surface. The selected power level (60 W) was sufficient to initiate plasma discharge without causing physical damage to the seed coat. At the same time, the relatively low gas flow rate facilitated extended residence time of reactive species close the seed surface. Figure 2 illustrates the emitted ROS and RNS via plasma device.

**Table 1:** Plasma process parameters for Cowpea seed treatment.

Parameter	Value	Description
Discharge power	60 W	Applied RF power to initiate plasma discharge
Frequency	13.56 MHz	Standard RF frequency for capacitive coupling
Gas type	Atmospheric air	Working gas used in the plasma chamber comprising of N₂ ~78%, O₂ ~21%, trace gases (Ar, CO₂) ~1% and water vapor (humidity) < 5%
Gas flow rate	1.5 sccm	Controlled by a mass flow controller
Chamber pressure	0.9 mbar	Maintained using a rotary vacuum pump
Exposure durations	1.0 min and 2.0 min	Selected to balance reactivity and avoid seed damage
Plasma configuration	Cylindrical plate capacitive RF reactor	Ensures uniform exposure over the seed surface
Seed positioning	Rotated cylindrical plate	Smooth treatment and prevents overheating

### Agronomic procedures and assessments

#### Wire house experiment

To define the most effective exposure time (dose) of plasma to be used in field conditions, a preliminary germinating test was performed under wire house. Cowpea seeds were procured from the Forage Crops Research Department, Agricultural Research Centre, Ministry of Agriculture & Land Reclamation, Egypt. Herein, the seeds of cowpea treated with three plasma times (1.0, 2.0 and 3.0 min, min), in addition to the control treatment (without plasma, 0.0 min). At the end of May 2022, five seeds per hole were planted in 40-hole plastic trays having (with diminutions of 6.0 × cm 4.5 cm × 4.5 cm), filled with soil and maintained under wire house conditions. At 2 weeks after planting (WAP), the seedlings were thinned to secure two seedlings per hole. Using a chlorophyll meter (SPAD–502, KONICAMINOLTA Inc., Tokyo), relative chlorophyll content was measured^45^ at 3 WAP. Next, at 4 WAP, seedlings were collected to assess the length and fresh weight.

#### Field experiment

At the experimental farm, Faculty of Agriculture Fayoum University, Fayoum, Egypt (29° 18’ N and 30° 56’ E), a model of a salt-affected soil area, cowpea plants were cultivated in two seasons of 2022 and 2023. The soil was a sandy loam with averages of 1.70 g cm⁻³, 41.0%, 12.2%, 2.21 cm h⁻¹, 0.62%, 4.40% and 7.9 for bulk density, total porosity, water-holding pores, hydraulic conductivity, organic matter, calcium carbonate, and pH, respectively. Single superphosphate (15.5% P_2_O_5_), 70 kg P_2_O_5_ ha^−1^ was incorporated during soil preparation. Next, according to soil analysis, mainly electrical conductivity (EC) the land could be naturally sectioned into three clusters of plots; low or no soil salinity (3.0 dS m⁻¹ EC), moderate soil salinity (5.5 dS m⁻¹ EC) and high soil salinity (7.0 dS m⁻¹ EC). Each plot sized 9.0 m^2^ involving 5.0 rows with 3.0 m in length and 0.60 m in width. Since the wire house study demonstrated that the plasma dose for 3.0 min showed seedling growth similar to 2.0 min, it was excluded from the field experiment. Therefore, cowpea seeds (cv. GIZA-18) were exposed to three plasma doses (0.0, 1.0, and 2.0 min) immediately before planting. Under the three salinity levels (3.0, 5.5 and 7.0 dS m⁻¹), seeds were sown on 25 and 12 June in the first and second seasons. Five seeds per hill were drilled on two sides of the ridge at a distance of 20.0 cm. At 5 WAP, plants received potassium sulfate (48% K_2_O) at a rate of 100 kg K_2_O ha^−1^. Plants were watered 12 day-interval via utilizing the furrow flood irrigation system. Irrigation water had 0.35 dS m⁻¹), and pH of 7.5.

Cowpea plants were harvested on 6th September 2022 and 25th August 2023. Plant samples were taken to measure plant height, number of branches per plant, number of leaves per plant, fresh weight of leaves per plant, and total forage yield per plant.

#### Experimental design and data analysis

The wire house experiment and field study experiment were tailored in a randomized complete block design using 4 replications. Both experimental obtained data were analyzed via performing the analysis of variance (ANOVA) as explained by Casella^46^, utilizing the program of COSTAT software, Version-6.303–2004.303. Based on Duncan’s multiple-range test^47^, differences among means of treatments were distinguished when the significance of F-test was established at *p* ≤ 0.05.

### In vitro evaluation

#### Chemical composition

The different experimental samples were oven-dried at 65 °C for 72 h, then ground in a cutter-type mill with a 1 mm screen. The samples were then subjected to proximate chemical analyses, including total nitrogen, crude fibers (CF), ether extract (EE), and ash according to AOAC^48^. Additionally, crude protein (CP) was calculated, and nitrogen-free extract (NFE) was calculated by difference. Furthermore, neutral detergent fiber (NDF), and acid detergent fiber (ADF) were determined^49^, using the Ankom200 (Ankom Technology Corp., Fairport, NY) filter bag technique.

#### Phytochemical screening

Qualitative phytochemical screening was carried out on the alcoholic extracts of cowpea. The presence of saponins and tannins were assessed^50^. Quantitative determination of total tannins was performed^51^. Total phenol content was estimated using the colorimetric method described by Snell and Snell^52^. Estimation of total saponins was conducted based on the method of Obadoni and Ochuko^53^ and Okwu and Ukanwa^54^.

#### Gas production experiment

An in vitro batch culture technique was applied as described by Meteab et al.^55^. The experiment was carried out in triplicate for each treatment. About 500 ± 3 mg of sample (according to the experimental design) was weighed into 120 ml incubation vessels. At least 3 vessels were included as a blank, and Alfalfa hay and concentrate were used in triplicate as the standard to generate the correction factor. Rumen fluid was obtained from adult sheep aged 1.5 to 2 years, fed clover hay for two weeks immediately after slaughter at Al-Marg slaughterhouse. The collected rumen fluid was mixed and squeezed through 4-layer cheesecloth into a bottle (2 L) with an O_2_-free headspace and maintained in an insulated container containing warm water 39 °C, then immediately transported to the laboratory.

Buffer solution was made up of 9.8 g NaHCO_3_, 2.44 g Na_2_HPO_4_, 0.57 g KCl, 0.47 g NaCl, 0.12 g MgSO_4_.7H_2_O, and 0.16 g CaCl_2_.2H_2_O per liter of distilled water. It is important to note that CaCl_2_ must be added only after all the other components have completely dissolved. During the warming and reducing step, urea is added to the buffer at a rate of 1.0 g/liter. Rumen fluid was mixed with buffer solution in a ratio of 1:4 (v/v) to use as a source of inoculum. Each vessel was filled with 50 ml of the incubation medium and dispensed anaerobically before being closed. The samples were then incubated at 39 °C for 24 h. Finally, the vessels were randomly distributed in the rack in the incubator and the tubes were swirling continuously.

Volumes of gas produced were measured after 24 h using a 100 ml glass syringe as described by Menke and Steingass^56^ and validated as outlined by Sarkwa et al.^57^. To calculate the accurate volume of gas produced, the formula 1 was used.

1 $${\mathrm{GP}}\left( {{\mathrm{ml/sample}}} \right)=({{\mathrm{V}}_{24}} - {\mathrm{G}}{{\mathrm{P}}_0}){\mathrm{x}}(({{\mathrm{F}}_{\mathrm{H}}}+{{\mathrm{F}}_{{\mathrm{CONC}}}})/2)$$

Where: V_24_: volume of gas produced after 24 h of incubation, GP_0_: volume of gas produced by the blank after 24 h of incubation, F_H_: volume of gas produced by standard hay/recorded gas production of standard hay, F_CONC_: volume of gas produced by standard concentrate/recorded gas production of standard concentrate.

After 24 h of incubation, the gas production was recorded. Then, the filtration process was performed on each of the 120 ml vessels using a filter bag (F57 Ankom). After the filtration process, the filter bags were dried at 105° C for 3 h in an oven to estimate residual DM, NDF, and ADF. The dry matter degradability (IVDMD), neutral detergent fiber degradability (IVDNDF), and acid detergent fiber degradability (IVDADF) were calculated as the difference between the weight of the incubated substrate and the weight of non-degraded residue at the end of incubation, according to formula 2^49^.

2 $${\text{IVD }}\left( \% \right){\text{ }}=~(({\mathrm{W}}1-{\text{ W}}2)){\text{ }}/{\text{ W}}1 \times {\text{ }}100$$

Where: W1: weight of the sample inside, W2: the true weight of the out sample.

The pH of rumen liquor was immediately recorded using a pH meter. Rumen liquor samples were analyzed to determine ammonia concentration (NH_3_) by Nessler’s method which modified by Szumacher-Strabel et al.^58^ and total volatile fatty acids (TVFAs) by steam distillation^59^.

#### *In vitro* data analyses

The experiment was designed as a factorial experiment (3 plasma doses × 3 salinity levels) in a completely randomized design. Data were statistically analyzed using the statistical analysis system SAS software^60^. Separation among means was carried out according to Duncan’s multiple-range test^47^ when the main factor was significant and *P* ≤ 0.05. The collected data were subjected to the analysis of variance in two ways, with the interaction analysis model according to the general linear model. The statistical model is presented in formula 3.

3 $${\text{Y ijk}}=~\upmu \,+\,{\mathrm{Ai}}\,+\,{\text{Bj }}+{\text{ }}({\text{AB ij}}\,{\mathrm{+}}\,{\mathrm{Eijk}}$$

Where, Y ijk: the K th Observation on the sample subjected to factors I and J, µ: general average; Ai: effect of the seed plasma exposure duration (i = 1,2 and3), Bj: effect of soil salinity level (j = 1,2 and 3), (AB)ij: effect of the interaction between factors i and j; Eijk: residual error on the ruminal liquid subjected to factors i and j.

## Results

### Cowpea seedling growth and yield traits

**Fig. 3:**
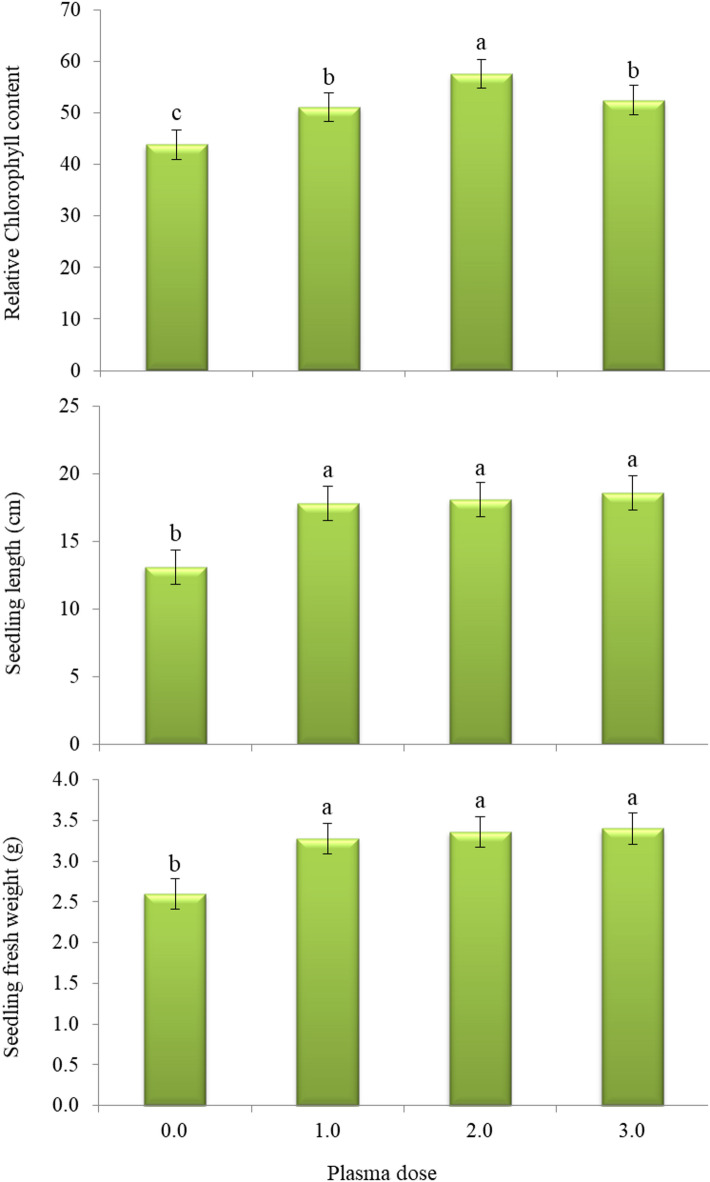
Relative chlorophyll content, length and fresh weight of cowpea cv. GIZA-18 seedlings sprouted from plasma-treated seeds at different doses (0.0, 1.0 and 2.0-minute, min). Varied letters within columns points out that there are significant differences at 0.05 level of probability. Means were distinguished employing Duncan’s multiple range test (*p ≤ 0.05*).

Concerning the findings observed from wire house experiment, plasma dose treatments exhibited distinctive changes in all seedling growth traits (Fig. 3). Cowpea seedlings sprouted form seeds that treated by plasma for 1.0, 2.0–3.0 min outperformed the control treatment (0.0 min) in relative chlorophyll content, seedling length and seedling fresh weight. The improvements were 16.2, 31.2 and 19.6% in relative chlorophyll content, 35.8, 38.2 and 41.9% in seedling length, and 26.2, 29.2 and 30.7% in seedling fresh weight owing to application of 1.0, 2.0–3.0 min, respectively, as comparing to the control treatment.

As for the field study, plasma treatment showed beneficial influence on cowpea cv. GIZA-18 yield attributes under non-salinized or salinized soil conditions in 2022 and 2023 seasons (Table 2). Obviously, treating seeds by plasma for 1.0 min and 2.0 min resulted in remarkable enhancements in yield and yield traits whether under normal or saline conditions. However, the increases in branches number plant^–1^ were more pronounced under non-saline soil in both seasons (with plasma dose of 2.0 min) as well as under moderate salinity (with plasma dose of 1.0 min) and high salinity (with plasma dose of 1.0–2.0 min) in the second, surpassing their counterpart control treatment (0.0 min). The maximal enhancements in number and fresh weight of leaves plant^–1^ were recorded with plasma dose of 2.0 min under non-saline soil and plasma dose of 1.0 min under moderate salinity in the first season. In the second season, plasma dose of 1.0 min was the efficient practice for enhancing number and fresh weight of leaves plant^–1^ under moderate and high salinity. In the first season, plasma dose of 2.0 min under non-saline soil and plasma dose of 1.0 min under moderate salinity were the efficient treatments for enhancing cowpea forage yield, outperforming the corresponding control treatment (without plasma, 0.0 min) by 111.2 and 47.0%, respectively. Under all salinity levels, plasma dose of 1.0–2.0 min exceeded the corresponding control treatment for elevating forage yield in the second season. Hence, the increases resulting with plasma dose of 1.0 min and 2.0 min amounted to 66.7 and 47.2% under non-saline soil, 254.1 and 146.1% under moderate salinity and 218.7 and 149.5% under high salinity, respectively.

**Table 2:** Influence of plasma dose on yield traits of Cowpea cv. GIZA-18 under different salinity levels in 2022 and 2023 seasons.

Treatment		Branches number plant^–1^	Leaves number plant^–1^	Leaves fresh weight plant^–1^ (g)	Forage yield plant^–1^ (g)
Season of 2022					
No-salinity	PD2	6.33a	55.4a	117.8a	304.5a
	PD1	5.58ab	46.9a-d	86.4a-c	223.6a-c
	PD0	3.83b-c	39.7b-e	67.5 cd	144.2c-e
Moderate salinity	PD2	4.50a-c	43.7a-d	76.5b-d	223.6a-c
	PD1	6.00a	54.4ab	104.7ab	283.2a
	PD0	4.58a-c	38.5c-e	61.0c-e	192.7bc
High salinity	PD2	3.08bc	17.0f	31.2ef	74.2ef
	PD1	2.91c	13.4f	26.8ef	61.9ef
	PD0	2.66c	10.8f	17.8f	35.8f
Season of 2023					
No-salinity	PD2	4.67a	17.8ab	39.8ab	106.9a
	PD1	3.67ab	19.7ab	45.8ab	121.0a
	PD0	2.67b-d	16.8ab	28.3bc	72.6b-d
Moderate salinity	PD2	3.00a-c	16.9ab	34.3bc	98.70ab
	PD1	4.00ab	25.8a	55.8a	142.0a
	PD0	1.67 cd	8.5b	15.8c	40.1 cd
High salinity	PD2	3.33a-c	19.9ab	33.6bc	78.6a-c
	PD1	3.67ab	26.3a	45.5ab	100.4ab
	PD0	1.33d	9.3b	14.1c	31.5d

No-salinity, moderate salinity and high salinity: soil salinity at 3.0, 5.5 and 7.0 dS m^−1^, respectively; PD0, PD1 and PD2: plasma dose of 0.0, 1.0, and 2.0 min, respectively. Varied letters of columns points out that there are significant differences at 0.05 level of probability. Means were distinguished employing Duncan’s multiple range test (*p ≤ 0.05*).

### *In vitro* evaluation

#### Cowpea forage chemical composition

Cowpea forage grown at low and high salinity conditions had significantly higher ash and CP content compared to those grown at moderate salinity (Table 3). Specifically, the ash content was 162.43 and 164.6 mg/g DM for low and high salinity, respectively, versus 154.27 mg/g DM at moderate salinity (*p* = 0.0002). Similarly, CP content reached 150.17 and 156.7 mg/g DM under low and high salinity, respectively, while moderate salinity recorded 139.37 mg/g DM (*p* = 0.0083). Conversely, OM content significantly decreased under both of low and high salinity compared to moderate salinity (*p* = 0.0002). Additionally, the highest salinity level caused a significant reduction in cell wall contents, including neutral detergent fiber (NDF; *p* = 0.0154) and acid detergent fiber (ADF; *p* = 0.0007), although no significant differences were observed between low and moderate salinity. In contrast, salinity had no significant effect on NFE (*p* = 0.3194) or CF content (*p* = 0.6291). Moreover, calculated energy parameters (GE, DE, ME and TDN) increased significantly with rising salinity levels (*P* < 0.0001), with clear differences among all three salinity levels.

**Table 3:** Effect of plasma dose (PD) on chemical composition of Cowpea cv. GIZA-18 under different salinity (S) levels.

Variable	No-salinity			Moderate salinity			High salinity			SE	*p* value		
	PD2	PD1	PD0	PD2	PD1	PD0	PD2	PD1	PD0		PD	S	PD×S
Ash (g/kg)	170.1^a^	159^dc^	158.2^dc^	150.5^e^	155.4^de^	156.9^dc^	162.4^bc^	166^ba^	165.4^ba^	1.2	0.8152	0.0002	0.0047
DM (g/kg)	928.5	924.7	926.3	929.1	922.9	929.7	925.1	929.4	927.4	2.3	0.4812	0.8918	0.2238
OM (g/kg)	829.8^e^	841^bc^	841.7^bc^	849.4^a^	844.6^ba^	843.1^bc^	837.6^dc^	834^de^	834.5^de^	1.2	0.8152	0.0002	0.0047
CF (g/kg)	354.8	397	359.2	362	379.5	353	373	301.9	383.4	12.8	0.9501	0.6291	0.1014
CP (g/kg)	157.8b^a^	144.4^bdc^	148.3^bdc^	133.9^d^	148.2^bdc^	136^d^	161.1^bac^	166.9^a^	142.1^dc^	2.8	0.0543	0.0083	0.0435
NFE (g/kg)	281.7	271.6	306.2	322.2	286.8	323.8	270	320.8	262.4	12.9	0.9418	0.3194	0.2274
NDF (g/kg)	563.1	549.5	554.2	568.6	608	51.72	526.5	520.2	458.5	12.9	0.0499	0.0154	0.3175
ADF (g/kg)	358.7	371.1	333.6	374.5	398.3	344.9	350.4	320.5	289.7	6.2	0.0021	0.0007	0.2047
GE (kcal/kg DM)	3480.41^f^	3503.545^ef^	3506.63^ef^	3554.03^bac^	3528.64^edc^	3523.87^ed^	3534.27^bdc^	3557.53^ba^	3570.91^a^	4.54	0.2795	≤ 0.0001	0.0137
DE (kcal/kg DM)	2645.11^f^	2662.69^ef^	2665.04^ef^	2701.06^bac^	2681.76^edc^	2678.14^ed^	2686.04^bdc^	2703.72^ba^	2713.89^a^	3.45	0.2797	≤ 0.0001	0.0137
ME (kcal/kg DM)	2168.99^f^	2183.4^ef^	2185.33^ef^	2214.87^bac^	2199.04^edc^	2196.07^ed^	2202.55^bdc^	2217.05^ba^	2225.39^a^	2.83	0.2796	≤ 0.0001	0.0137
TDN (%)	59.99^f^	60.39^ef^	60.44^ef^	61.26^bac^	60.82^edc^	60.74^ed^	60.92^bdc^	61.32^ba^	61.55^a^	0.07	0.2929	≤ 0.0001	0.014

No-salinity, moderate salinity and high salinity: soil salinity at 3.0, 5.5 and 7.0 dS m^− 1^, respectively; PD0, PD1 and PD2: plasma dose of 0.0, 1.0, and 2.0 min, respectively. DM: dry matter, OM: organic matter, CF: crude fiber, CP: crude protein, NFE: nitrogen-free extract, NDF: neutral detergent fiber, ADF: acid detergent fiber, GE: gross energy, DE: digestible energy, ME: metabolizable energy and TDN: total digestible nutrients. Varied letters within rows points out that there are significant differences at 0.05 level of probability. Means were distinguished employing Duncan’s multiple range test (*p ≤ 0.05*).

Concerning the plasma exposure duration effect, the data of Table 3 showed that seed plasma treatment did not significantly affect ash, OM, CF, CP, and NFE contents, nor did it affect the calculated energy values, including GE, DE, ME, and TDN (*P* > 0.05). However, seeds exposed to cold plasma for 1–2 min produced plants with significantly higher NDF and ADF content compared to untreated seeds. Specifically, the NDF content was 559.23 and 552.73 mg/g DM for the 1- and 2-minute treatments, respectively, versus 509.97 mg/g DM for the control. Likewise, ADF content reached 363.3 and 361.2 mg/g DM for treated seeds, compared to 322.73 mg/g DM for the untreated seeds, with P-values of 0.0021 for NDF and 0.0499 for ADF.

A significant interaction (*P* < 0.05) between plasma treatment duration and salinity levels was observed for ash, OM, CP, and calculated Energy Values (GE, DE, ME, TDN). Plants grown from seeds treated with plasma for 2 min under low salinity showed the highest ash and the lowest OM content. Conversely, plasma dose of 2.0 min under moderate salinity produced plants with the lowest ash content and higher OM values. The highest CP content (166.9 g/kg) was recorded for plants produced from plasma dose of 1.0 min under high salinity, followed by 161.1 g/kg for 2.0 min under the same salinity level. The lowest CP content (133.9 g/kg) was observed at 2.0 min under moderate salinity. For calculated energy values, the interaction between plasma exposure and salinity was statistically significant (*p* = 0.0137). At the highest salinity level, plasma treatments of both 1.0 and 2.0 min enhanced energy parameters compared to the control (0.0 min). However, at low salinity levels, plasma doses, particularly at 2.0 min, slightly reduced energy content relative to control (0.0 min).

#### Phytochemical screening

The qualitative screening of anti-nutritional factors (ANFs) in cowpea forage (Table 4) revealed that salinity alone did not affect tannin intensity, as plants derived from both untreated and treated seeds showed the same qualitative levels across all salinity conditions. In contrast, saponin intensity increased progressively in plants grown from untreated seeds as salinity levels rose, with moderate (++) levels at low salinity and strong (+++) levels at both moderate and high salinity.

**Table 4:** Effect of plasma dose on qualitative screening of the anti-nutritional factors of Cowpea cv. GIZA-18 under different salinity levels.

Variable	No-salinity			Moderate salinity			High salinity		
	PD2	PD1	PD0	PD2	PD1	PD0	PD2	PD1	PD0
Total tannins	+	+	++	+	+	++	+	+	++
Total saponin	++	+	++	++	+	+++	++	+	+++

No-salinity, moderate salinity and high salinity: soil salinity at 3.0, 5.5 and 7.0 dS m^− 1^, respectively; PD0, PD1 and PD2: plasma dose of 0.0, 1.0, and 2.0 min, respectively. + indicates presence (low intensity), ++ indicates moderate presence and +++ indicates high presence.

With respect to plasma doses, under control conditions (0 min plasma), tannins consistently showed moderate (++) levels, irrespective of salinity level. However, plasma doses of 1.0 and 2.0 min markedly reduced tannin intensity, with only low (+) levels across all salinity levels. For saponins, a notable increase was observed in control treatments (0.0 min) as salinity increased, with strong (+++) levels at moderate and high salinity conditions, and moderate (++) levels at low salinity conditions. Seed plasma treatment for 1.0 min drastically reduced saponin intensity to low levels (+) across all salinity levels. Interestingly, when plasma duration was extended to 2.0 min, saponin intensity increased slightly to moderate (++) levels, irrespective of salinity level.

The quantitative analysis of anti-nutritional and phenolic compounds in cowpea forage (Table 5) highlighted both individual and interactive effects of salinity and plasma exposure duration. The data showed that tannin concentration was lowest under moderate salinity (7.97 mg/100 g DM), while both non-salinity and high salinity showed slightly higher values (8.64 and 8.49 mg/100 g DM, respectively). However, moderate and high salinity increased total saponin content (1.08 and 1.44 g/100 g DM, respectively) compared with the non-saline condition (0.63 g/100 g DM). Total phenols remained relatively stable across salinity treatments, with values between 0.91 and 0.92%.

**Table 5:** Effect of plasma dose (PD) and salinity (S) level and their interaction (PD×S) on total tannins, total saponins and total phenols concentration of Cowpea cv. GIZA-18.

Variable		Total tannins (mg/100 g DM)	Total saponin (g/100 g DM)	Total phenol (%)
PD				
PD2		7.51	0.79	0.91
PD1		8.04	0.80	0.90
PD0		9.54	1.55	0.94
S				
No-salinity		8.64	0.63	0.93
Moderate salinity		7.97	1.08	0.92
High salinity		8.49	1.44	0.92
PD×S				
No-salinity	PD2	8.11	0.33	0.91
	PD1	8.26	0.69	0.89
	PD0	9.55	0.87	0.98
Moderate salinity	PD2	7.16	1.06	0.92
	PD1	7.48	1.10	0.91
	PD0	9.26	1.07	0.92
High salinity	PD2	7.26	0.98	0.90
	PD1	8.38	0.62	0.92
	PD0	9.82	2.71	0.93

No-salinity, moderate salinity and high salinity: soil salinity at 3.0, 5.5 and 7.0 dS m^− 1^, respectively; PD0, PD1 and PD2: plasma dose of 0.0, 1.0, and 2.0 min, respectively. DM: dry matter.

Plasma treatment had a pronounced effect on tannins, which declined markedly with increasing exposure duration. Control plants (0 min) had the highest tannin concentration (9.54 mg/100 g DM), whereas seeds treated for 1.0 and 2.0 min produced plants with lower tannin levels (8.04 and 7.51 mg/100 g DM, respectively). Saponin concentration also decreased under plasma treatment, from 1.55 g/100 g DM in control plants to 0.80 and 0.79 g/100 g DM after 1 and 2 min, respectively. In contrast, plasma had little effect on total phenols, which remained relatively stable (from 0.90 to0. 94%).

The interaction between plasma dose and salinity revealed that plasma treatment mitigates the salinity effect in tannins and saponins. For instance, under high salinity conditions, seed treatment for 2 min reduced tannin and saponin levels in the produced plants (7.26 mg/100 g DM and 0.98 g/100 g DM, respectively) compared with untreated seed (9.82 mg% and 2.71 g/100 g DM). Similarly, under moderate salinity, seed treatment for 2 min lowered tannins (7.16 mg/100 g DM) relative to untreated seed (9.26 mg/100 g DM). In contrast, total phenols showed only slight variation across all treatments, with values ranging from 0.89 to 0.98%.

#### Degradability parameters

**Fig. 4:**
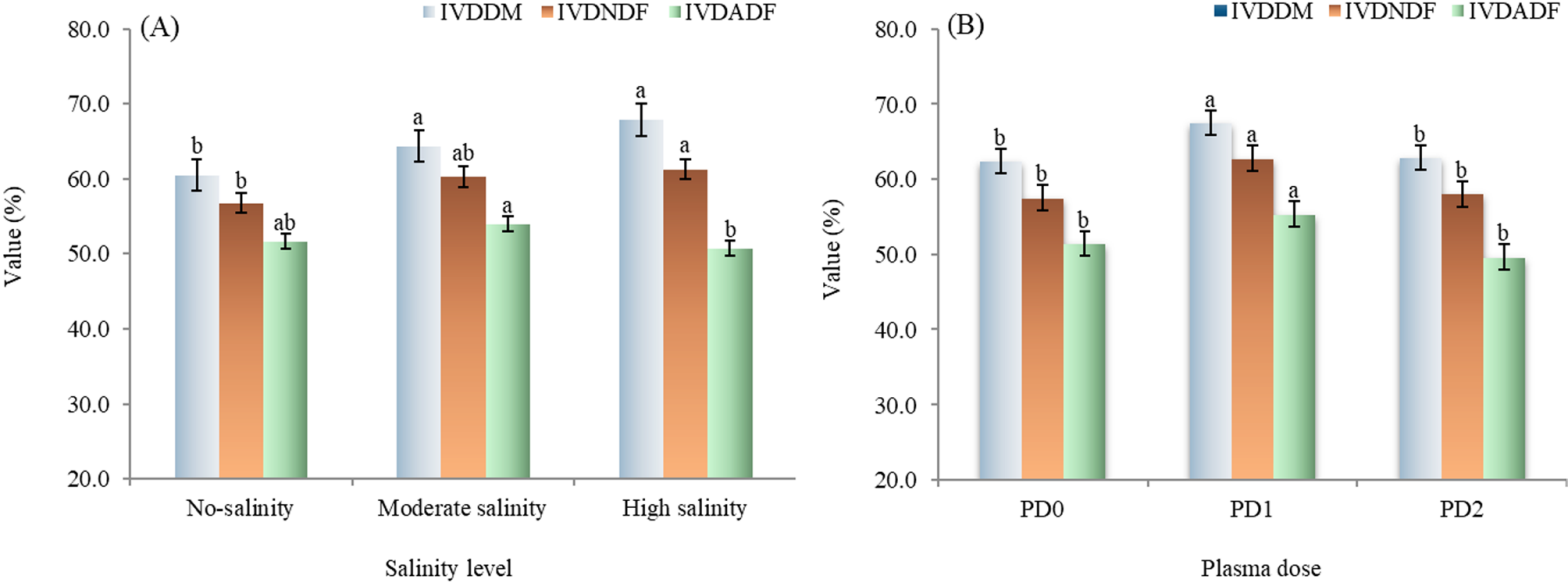
The main effects of salinity level (**A**) and plasma dose (**B**) on nutrients degradability (%) of cowpea cv. GIZA-18. No-salinity, moderate salinity and high salinity: soil salinity at 3.0, 5.5 and 7.0 dS m^− 1^, respectively; PD0, PD1 and PD2: plasma dose of 0.0, 1.0, and 2.0 min, respectively. IVDDM: in vitro degradability of dry matter, IVDNDF: in vitro degradability of neutral detergent fiber and IVDADF: in vitro degradability of acid detergent fiber. Varied letters of columns points out that there are significant differences at 0.05 level of probability. Means were distinguished employing Duncan’s multiple range test (*p ≤ 0.05*).

Salinity had a significant impact on in vitro degradability of dry matter (IVDDM), neutral detergent fiber (IVDNDF), and acid detergent fiber (IVDADF) (Table 6; Fig. 4). Cowpea plants grown under moderate and high salinity recorded significantly higher IVDDM compared to those grown at low salinity levels (*p* = 0.0016). Also, plants grown at the high salinity level had significantly higher values of IVDNDF compared to those grown at low salinity levels (*p* = 0.0319). In contrast, IVDADF was significantly higher under moderate salinity compared with high salinity (*p* = 0.049).

**Table 6:** Effect of plasma dose (PD) on in vitro nutrients degradability and gas production (GP) parameters of Cowpea cv. GIZA-18 under different salinity (S) levels.

Variable		Nutrients degradability (%)			Gas production			
		IVDDM	IVDNDF	IVDADF	GP (ml/g DM)	GP (ml/g OM)	GP (ml/g NDF)	GP (ml/g ADF)
No-salinity	PD2	59.40^c^	57.64^a^	49.81^dc^	146.06	176.00	240.82^b^	378.16^d^
	PD1	71.27^a^	63.52^a^	60.01^a^	143.15	170.22	240.92^b^	356.68^de^
	PD0	50.84^d^	49.20^b^	45.13^d^	153.36	182.20	256.29^b^	425.82^bc^
Moderate salinity	PD2	61.88^bc^	57.72^a^	47.95^dc^	152.30	179.30	248.91^b^	377.91^d^
	PD1	65.20^bac^	61.82^a^	57.91^ba^	137.68	163.02	208.96^c^	319.01^e^
	PD0	66.03^bac^	61.16^a^	56.13^ba^	164.56	195.18	295.75^a^	443.59^ba^
High salinity	PD2	67.31^ba^	58.65^a^	51.08^c^	149.09	177.99	261.92^b^	393.60^dc^
	PD1	66.06^bac^	62.97^a^	48.05^dc^	146.70	175.90	262.07^b^	425.36^bc^
	PD0	70.30^a^	62.18^a^	53.05^bc^	147.61	176.87	298.60^a^	472.51^a^
**SE**		2.34	2.11	1.62	5.2	6.21	9.23	14.41
p value	PD	0.0185	0.0062	0.0003	0.0173	0.0179	≤ 0.0001	≤ 0.0001
	S	0.0016	0.0319	0.049	0.5804	0.826	0.0011	0.0001
	PD×S	≤ 0.0001	0.0062	≤ 0.0001	0.1764	0.1834	0.0058	0.0232

No-salinity, moderate salinity and high salinity: soil salinity at 3.0, 5.5 and 7.0 dS m^− 1^, respectively; PD0, PD1 and PD2: plasma dose of 0.0, 1.0, and 2.0 min, respectively. IVDDM: in vitro degradability of dry matter, IVDNDF: in vitro degradability of neutral detergent fiber and IVDADF: in vitro degradability of acid detergent fiber; DM: dry matter, OM: organic matter, NDF: neutral detergent fiber and ADF: acid detergent fiber. Varied letters of columns points out that there are significant differences at 0.05 level of probability. Means were distinguished employing Duncan’s multiple range test (*p ≤ 0.05*).

Cowpea forage produced from seeds exposed to plasma for 1.0 min showed significantly higher IVDDM, IVDNDF, and IVDADF compared with both untreated (0.0 min) and 2.0 min dose (*p* = 0.0185, 0.0062, and 0.0003, respectively), with no significant differences between the 0.0 and 2.0 min treatment (Fig. 4).

A significant interaction between plasma exposure and salinity was detected for IVDDM (*P* < 0.0001), IVDNDF (*p* = 0.0062), and IVDADF (*P* < 0.0001). The data showed that IVDDM increased with salinity under control conditions (0 min), rising from 50.84% at low salinity to 70.30% at high salinity. A similar trend was observed at 2.0 min plasma, where values increased from 59.40% at low salinity to 67.31% at high salinity. However, this trend reversed for 1.0 min treatment, IVDDM decreased from 71.27% at low salinity to 66.06% at high salinity. The highest IVDDM was recorded for a 1.0 min dose under low salinity and the 0.0 min dose under high salinity, whereas the lowest value was observed for the 0.0 min dose under low salinity. For IVDNDF, the lowest significant value was recorded for the 0.0 min under low salinity, with no significant differences among the other combinations. However, the highest significant value of IVDNDF occurred for the 1.0 min dose under low salinity, while the lowest significant value was again noted for the control (0.0 min) under low salinity.

#### Gas production parameters

**Fig. 5:**
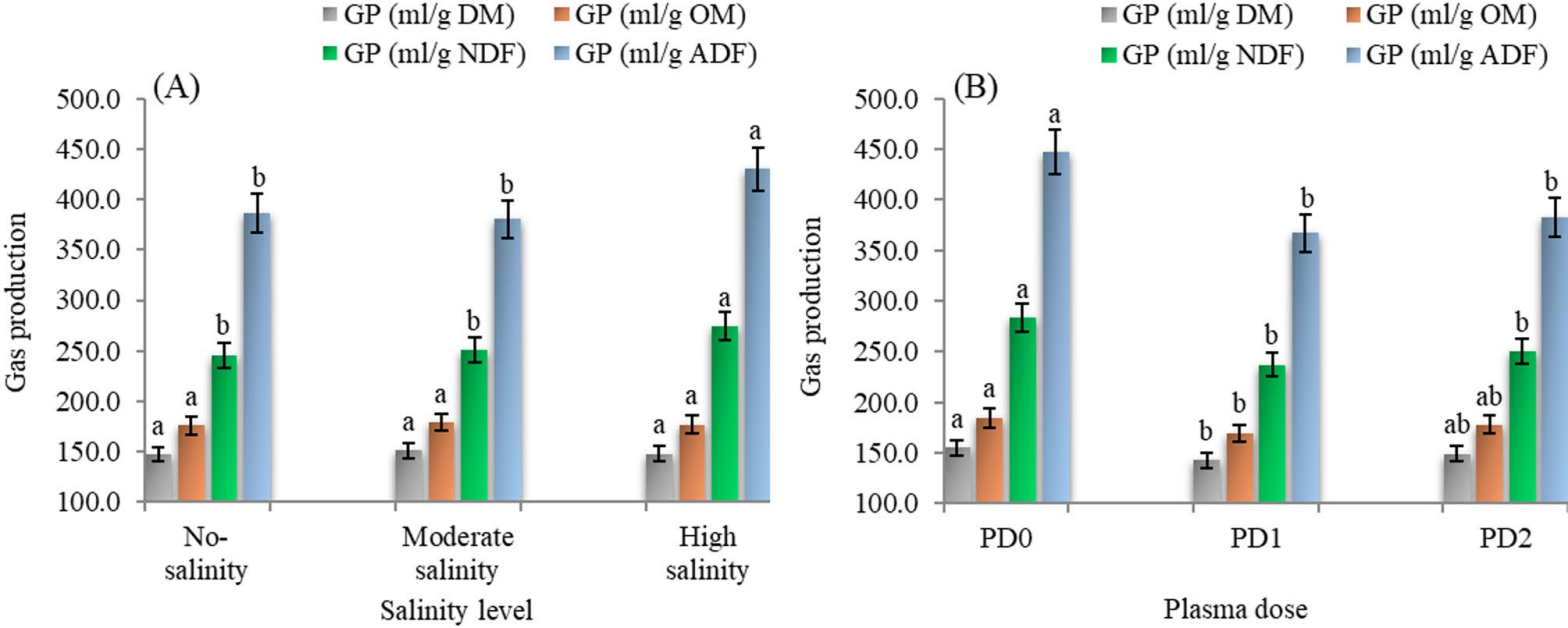
The main effects of salinity level (**A**) and plasma dose (**B**) on gas production (based on DM, OM, NDF and ADF) of cowpea cv. GIZA-18. No-salinity, moderate salinity and high salinity: soil salinity at 3.0, 5.5 and 7.0 dS m^− 1^, respectively; PD0, PD1 and PD2: plasma dose of 0.0, 1.0, and 2.0 min, respectively. DM: dry matter, OM: organic matter, NDF: neutral detergent fiber and ADF: acid detergent fiber. Varied letters of columns points out that there are significant differences at 0.05 level of probability. Means were distinguished employing Duncan’s multiple range test (*p ≤ 0.05*).

The data presented in Table 6; Fig. 5 indicated that salinity level had no significant effect on gas production per gram of dry matter (GP/g DM; *p* = 0.5804) or gas production per gram of dry matter (GP/g OM; *p* = 0.826) after 24 h of in vitro fermentation of cowpea forage. However, plants grown under high salinity exhibited significantly higher gas production per gram of NDF (GP/g NDF; *p* = 0.0011) and gas production per gram of ADF (GP/g ADF; *p* = 0.0001) compared to those grown under low and moderate salinity.

Plasma doses significantly influenced gas production (Fig. 5). Plasma dose of 1.0 min significantly reduced GP/g DM (*P* = 0.0173) and GP/g OM (*P* = 0.0179) compared to control (0 min), while no significant differences were observed between 0.0 and 2.0 min doses. A similar inhibitory pattern was observed in fiber-associated gas production; the cowpea plants treated with plasma for 1.0 and 2.0 min significantly reduced GP/g NDF and GP/g ADF compared to untreated seeds (*P* < 0.0001).

A significant interaction between plasma treatment and salinity was detected for GP/g NDF (*p* = 0.0058) and GP/g ADF (*p* = 0.0232). Under high salinity (EC 7.0), untreated plants recorded the highest GP values (298.60 ml/g NDF and 472.51 ml/g ADF), while 1.0 min dose reduced these values by 12.2% (262.07 ml/g NDF) and 10.0% (425.36 ml/g ADF), respectively. The 2.0 min dose showed intermediate values. No significant interactions were found for GP/g DM (*p* = 0.1764) or GP/g OM (*p* = 0.1834).

#### Fermentation parameters

**Fig. 6:**
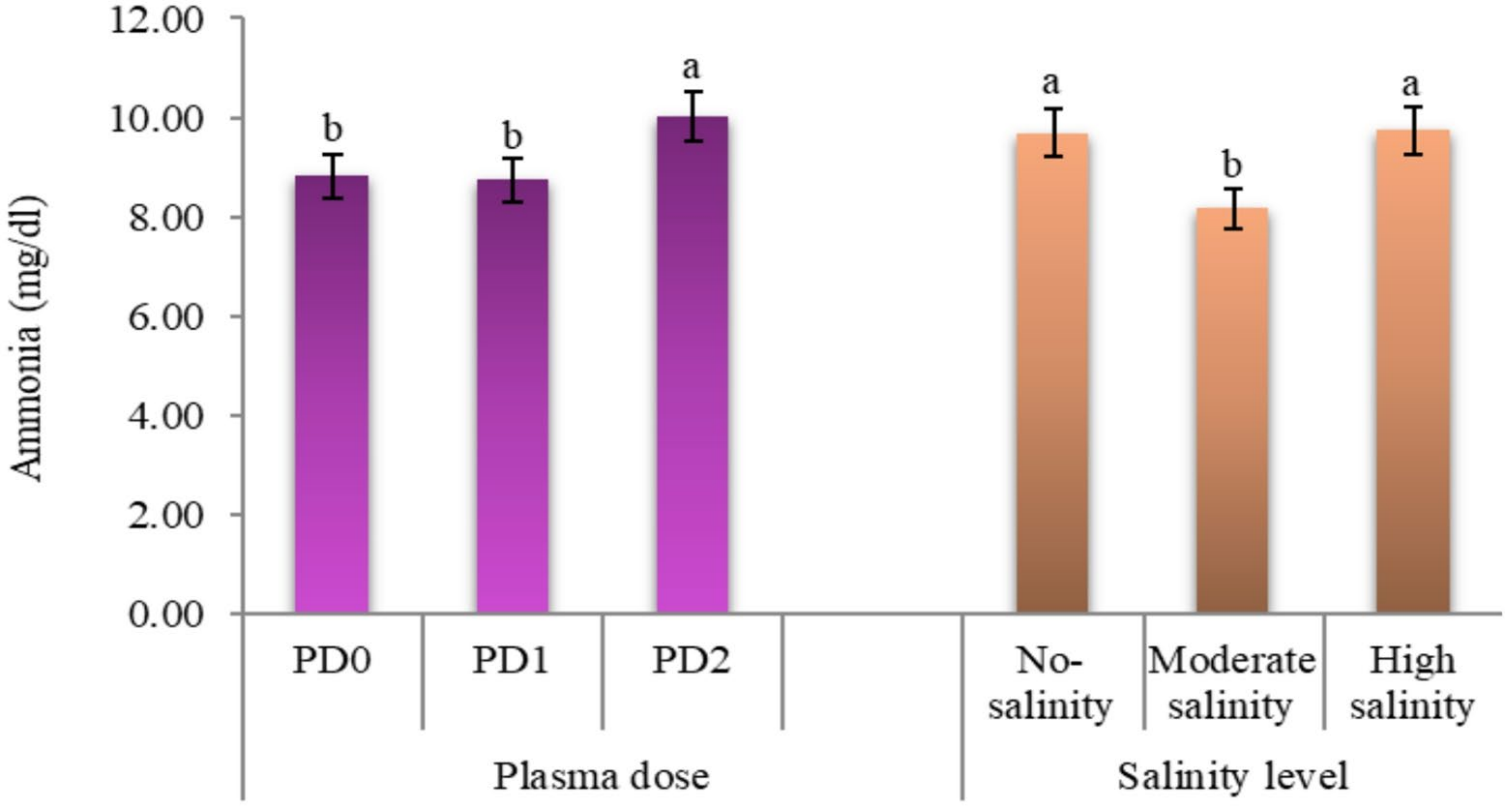
The main effects of plasma dose and salinity level on fermented forage expressed in ammonia for cowpea cv. GIZA-18. No-salinity, moderate salinity and high salinity: soil salinity at 3.0, 5.5 and 7.0 dS m^− 1^, respectively; PD0, PD1 and PD2: plasma dose of 0.0, 1.0, and 2.0 min, respectively. Varied letters of columns points out that there are significant differences at 0.05 level of probability. Means were distinguished employing Duncan’s multiple range test (*p ≤ 0.05*).

The data in Table 7 showed that plants grown under low and high salinity levels recorded significantly higher (*P* ≤ 0.0001) ammonia level compared to those grown under moderate salinity levels **(**Fig. 6**)**. Moreover, the data showed significantly higher pH values (*p* = 0.0432) were recorded for low and moderate salinity compared to the high salinity level, with no significant difference between low and moderate salinity. On the contrary, no significant effect for VFA concentration was recorded due to salinity effect (*p* = 0.3418).

**Table 7:** Effect of plasma dose on in vitro fermentation of Cowpea cv. GIZA-18 under different salinity levels.

Variable		pH value	Ammonia (mg/dl)	Total volatile fatty acids (meq/dl)
No-salinity	PD2	6.68	11.91^e^	9.28^a^
	PD1	6.67	8.82^d^	7.95^b^
	PD0	6.60	8.33^b^	7.85^b^
Moderate salinity	PD2	6.63	9.76^c^	9.17^a^
	PD1	6.65	6.69^a^	8.42^ba^
	PD0	6.65	8.04^d^	8.28^ba^
High salinity	PD2	6.58	8.39^d^	7.83^b^
	PD1	6.60	10.71^d^	8.67^ba^
	PD0	6.52	10.11^cb^	8.15^ba^
SE		0.04	0.29	0.35
p value	PD	0.3208	≤ 0.0001	0.0857
	S	0.0432	≤ 0.0001	0.3418
	PD×S	0.7738	≤ 0.0001	0.0443

No-salinity, moderate salinity and high salinity: soil salinity at 3.0, 5.5 and 7.0 dS m^− 1^, respectively; PD0, PD1 and PD2: plasma dose of 0.0, 1.0, and 2.0 min, respectively. Varied letters of columns points out that there are significant differences at 0.05 level of probability. Means were distinguished employing Duncan’s multiple range test (*p ≤ 0.05*).

Concerning plasma duration, plants grown from seeds exposed to 2.0 min had significantly higher ammonia concentrations compared to 1.0 min dose, which did not differ significantly from each other (*P* < 0.0001). On the contrary, plasma treatment did not affect substantially both pH value (*p* = 0.3208) and VFA concentration (*p* = 0.0857).

A significant interaction effect between seed plasma exposure duration and salinity was observed for ammonia concentration (*P* = 0.0001) and total VFA production (*p* = 0.0443); in contrast, no significant effect was recorded for pH value (*p* = 0.7738). The highest ammonia concentration was recorded for 2.0 min dose under low salinity, while the lowest concentration was observed in 1.0 min dose under moderate salinity. Concerning total VFA production, the interaction effect showed that the highest significant total VFA concentrations were observed for 2.0 min dose under low and moderate salinity, while the lowest total VFA concentrations were observed in the control (0.0 min) under low salinity and 2.0 min dose under high salinity.

## Discussion

### Cowpea growth and productivity

Ensuring good germination and vigorous seedlings is the key to sustainable crop production in saline soils. Plasma technology could participate in this matter as chlorophyll concentration, length and weight of cowpea seedlings were improved by application of different doses of plasma. Improvements in seedling growth have been observed using plasma treatment due to its chemical functionalization and etching influences with modulation of the seed surface and increase of its wettability and permeability^61^. Plasma enhanced seed germination^62,63^ and abiotic stress tolerance^61^, thus, improving crop productivity. Plasma has an influential role in synthesis of plant hormones in seeds and seedlings^64,65^. In this concern, seed germination was stimulated with application of NTP application, due to elevating the activity of hydrolytic enzyme and gibberellin level^66^. Auxins and cytokinin levels were up-regulated in seeds after subjecting to NTP^67^.

Nevertheless, the available information on the possibility of using plasma to relieve stress caused by salinity is still insufficient. Therefore, the present work endeavored to highlight the possible significant action of plasma dose for enhancing the stress tolerance in cowpea grown in salt-affected soils. According to the obtained field study findings, the ameliorative influences of plasma on forage yield and yield attributes of cowpea cv. GIZA-18 appeared under different levels of salinity, specifically under non-saline soil (EC of 3.0 dS m⁻¹) and with moderate salinity (EC of 5.5 dS m⁻¹). it has been proven that increased salt levels in growing medium not only result in ionic toxicity but also reduce the opportunity of roots to absorb sufficient moisture and nutrients, causing multiple stresses that induce oxidative injuries, hence weakening plant growth^68–70^. These oxidative stress types lead up to physiological dysfunctions^71–73^, and ultimately, negatively impact crop productivity and quality^74,75^. On the contrary, the impacts of salt toxicity are reduced with application of plasma, as it plays a crucial role in the activation of phenylalanine ammonia lyase and peroxidase^76^. Salinity is known to increase the level of malondialdehyde (MDA), leading in peroxidation of lipids with damage to the plant cell membranes^77^. Interestingly, NTP treatment decreased the level of MDA while stimulating germination and seedling development, indicating that timely subjecting seeds to plasma could preserve the integrity of membrane by motivating the antioxidant mechanism^61,78^. Morphological and biochemical modifications, more hydrophilic seeds, and increased tolerance to water deficiency and metal toxicity were recorded due to plasma application. Under harsh conditions, plasma has demonstrated tolerance to oxidative stress with improved water and nutrient uptake^79^. Recently, researches proved that plasma-treated seeds exhibited high rates of germination and seedling growth, which was associated with high levels of enzymatic activities and phytohormones, while improving stress tolerance and crop productivity^31,80^. Further, wheat seedlings sprouted from NTP-treated seeds had higher abscisic acid concentration than non-treated seeds^81^. This may interpret the potential action of plasma in relieving the salt stress, as abscisic acid regulates water balance and stomatal conductivity in addition to its inductive role in stress tolerance-related genes.

### In vitro evaluation

#### Cowpea forage chemical composition

Increased ash content due to salinity effect is likely an expression of increased mineral deposition in plant tissue due to saline stress, and this is frequently associated with ion absorption and osmotic adjustment. The observed increase in ash content reflects enhanced accumulation of inorganic ions such as Na⁺ and Cl⁻ in plant tissues, which is a well-recognized adaptive mechanism in salt-tolerant and moderately tolerant grasses to maintain osmotic balance^82^. On the other hand, a decrease in OM content may be because of impaired photosynthetic efficiency and reduced biosynthesis of organic comp ounds due to oxidative stress imposed by salinity^83^. A similar trend was observed by Saudy et al.^32^, who observed a significant reduction in OM contents in cowpea plants parallel to salinity increases. Fiber fractions (NDF and ADF) also recorded significant reductions under the high salinity level, suggesting that salt stress negatively affects structural carbohydrate synthesis and nitrogen metabolism. This agrees with previous reports that salinity stress suppresses cell wall development and protein synthesis^84^. Moreover, CP content increased at high salinity (*p* = 0.0083), which may be attributed to the abiotic stress-induced accumulation of nitrogenous compounds or stress-related proteins^85^. Additionally, Hasegawa et al. and Hussin et al.^86,87^ attributed the rise in crude protein (CP) concentration under saline conditions to two main factors: (i) a concentration effect resulting from reduced deposition of structural carbohydrates, and (ii) an increased synthesis of nitrogen-containing osmo-protective compounds and stress-related proteins that contribute to cellular protection and maintain ion homeostasis.

Energy content like GE, DE, ME and TDN increased as salinity increased (*P* ≤ 0.0001) which can be attributed to the reduction of fibers as NDF and ADF and the increase in CP content, this is not always reflective of better nutrient quality. Opposite trend was observed by Saudy et al.^32^, who observed a significant reduction in calculated feeding value as GE, DE, ME and TDN in cowpea plants parallel to salinity increases.

Concerning plasma effect, the non-significant (*P* > 0.05) differences in most of the parameters of chemical composition parameters such as Ash, OM, CF, CP, and NFE as well as the calculated energy values, i.e. GE, DE, ME and TDN due to the plasma doses suggest that this treatments had no adverse effects on the chemical composition of the grown cowpea plants. This aligns with Saudy et al.^32^, who observed no adverse effects of plasma treatment (0.0 to 2.0 min) on ash, OM, CP, NDF, or energy values in cowpea forage under saline. In contrast, previous work by Li et al.^88^ reported that seed exposed to cold plasma enhanced metabolic activity and increased synthesis of protective compounds under plasma treatment. Additionally, NDF and ADF content increased significantly (*p* = 0.0021 and 0.0499) in response to cold plasma treatment for 1 and 2-minute treatments. This indicates a potential modification of cell wall constituents as a result of cold plasma treatment, possibly linked to enhanced activity of enzymes, which modulate cellulose and hemicellulose restructuring^89^ (Le Gall et al. 2015). The present observations align with previous findings by Saudy et al.^32^, who observed significant increase in NDF and ADF for plants grown from seeds treated with cold plasma compared to control as well as align with findings of Li et al.^88^, suggesting that plasma treatment can enhance carbohydrate metabolism, thereby improving stress tolerance mechanisms. This may affect the digestibility and structural integrity of plant tissues.

Plasma and salinity interaction effects were statistically significant for Ash, OM, CP and calculated energy content (GE, DE, ME, and TDN), their combined effect impacts plant metabolism to a greater extent than single effects. The presented results suggest that cold plasma seed exposure for 1–2 min may reduce Ash accumulation and enhance organic content at moderate salinity, Notably, Saudy et al.^32^ reported that plasma treatment (2.00 min) reduced ash content by 12.5% and increased OM by 8% at high salinity (EC7.0) compared to untreated controls, confirming plasma’s role in counteracting salinity induced mineral accumulation. Additionally, cold plasma treatment enhances protein under severe salinity, possibly via improved nitrogen metabolism or stress adaptation. These results suggest that cold plasma enhances stress tolerance mechanisms. Conversely, the absence of an interaction effect on CF, NDF, and NFE content suggests that these traits are more affected by salinity per se and not by its interaction effect with plasma treatment.

#### Phytochemical screening

The data showed that salinity levels had a minimal effect on the determined anti-nutritional factors (tannins, saponins, and total phenols), although it is part of the plant’s adaptive defense mechanism. In contrast, the results of phytochemical screening show notable decreases in tannins and saponins due to seed exposure to cold plasma, qualitatively and quantitatively. This suggests that plasma exposure, even at minimal durations (1.0 min), effectively reduced tannin and saponins accumulation. Moreover, tannin levels did not differ between the 1-minute and 2-minute plasma treatments, indicating a possible threshold effect beyond which further plasma exposure did not enhance tannin degradation. This agrees with research that plasma-generated reactive oxygen and nitrogen species (RONS) break down phenolic compounds such as tannins through the oxidation of their molecular structures^90,91^. This reduction is approximately stable across salinity levels, indicating that plasma’s effectiveness does not depend on salt stress, rendering it an effective technique for tannins and saponins alleviation in the cultivated plants.

However, longer plasma exposure may interact with salinity, partially reversing salinity effects, particularly in the case of saponins. The partial increase in saponin levels may reflect a complex interaction between the oxidative effects of plasma and salinity-induced stress responses that stimulate the synthesis of secondary metabolites^92^. This reduction in ANFs supports the potential of plasma treatment to degrade tannin precursors or inhibit their biosynthesis^93^. Notably, there was a slight increase in saponin levels at 2 min compared to 1 min, likely due to oxidative stress reactivating saponin pathways during longer exposures. This suggests that plasma treatment primarily targets specific secondary metabolites such as tannins and saponins, while the overall phenolic content remains relatively stable. Furthermore, under plasma treatment, the effects of salinity appear diminished, indicating that plasma may mitigate the tannin inducing impacts of salt stress.

#### Degradability parameters

The results showed a significant increase in IVDDM was measured within plants grown under moderate and high salinity (EC5.5 and EC7.0). Enhanced in vitro dry matter degradability under moderate and high salinity has been linked to changes in cell wall composition, including reduced lignification and alterations in neutral detergent fiber fractions. These modifications improve microbial accessibility during rumen fermentation^94,95^. Additionally, the higher ash content of such plants may lead to an increased production of soluble fractions, potentially resulting in greater dry matter degradability. The increase in in vitro degradability of NDF under high salinity compared to low salinity (EC7.0 vs. EC3.0) may be explained by physiological and biochemical responses to salinity stress. Salinity induces osmotic stress, and plants may accumulate osmolytes, which are soluble sugars and proteins; osmolytes help keep cellular turgor and help protect cellular structures. According to Jayawardhane et al.^96^, cowpea plants under salinity stress presented higher levels of soluble sugars and proteins, which could have been beneficial for degradability. Also, Salinity can modify the structure of plant cell walls by decreasing their lignin concentration or modifying the ratio of structural carbohydrates, making the cell wall more sensitive to microbial degradation^97^. While studies looking specifically at cowpea are scarce, other legumes have shown similar trends, lending credence to this idea, as demonstrated by Neves et al.^98^ in soybean, where salt stress inhibited root growth and increased lignification. The responses from our study suggest that salinity leads to higher forage quality but lower forage quantity. This reduction in yield may be somewhat offset by improved digestibility and nutrient density. However, the increase in quality cannot fully compensate for the negative effects of decreased biomass production^99^. Therefore, salinity should be viewed primarily as a limitation on forage production capacity, rather than on its nutritional value, especially when salt-adapted species are cultivated within their tolerance limits.

Concerning the plasma duration effect, the data illustrated that seed plasma treatment for 1 min significantly increased IVDDM, IVDNDF, and IVDADF compared to those grown from seed treated for 2 min and untreated seeds, with no significant differences between 0 and 2-minute treatment. The results of acid detergent fiber degradability (ADFD) align with the findings of Saudy et al.^32^, indicating that a 1-minute plasma exposure increased ADFD by 17% under high salinity conditions (EC7.0). The observed increase in dry matter degradability can be attributed to non-thermal plasma treatment, which is known to induce surface etching, partial depolymerization of complex carbohydrates, and oxidation of lignocellulosic bonds. These processes enhance substrate accessibility for rumen microbes^100,101^. Additionally, the significant increases in nutrient degradability of plants grown from plasma-treated seeds may be due to the minor changes initiated in the cell wall structure and composition by plasma treatment. A direct effect of abiotic stressors like plasma exposure on cell wall dynamics was observed^88,89^. The improved degradability of NDF and ADF may also be attributed to higher cellulose content, as cellulose is quite fermentable^32^.

The interaction between plasma exposure time and salinity level had a significant effect, particularly on IVDDM and IVDADF. This interaction indicates that the efficacy of plasma treatment depends on salinity levels. The greatest improvement in IVDDM occurred at EC3.0, where values increased from 50.84% (untreated seeds) to 71.27% (seeds treated for 1 min). Similarly, IVDADF showed a notable increase at the same salinity level (60.01% baseline, improving with 1-minute plasma exposure). However, at higher salinity.

(EC7.0) with a 2.0 min exposure, no significant improvement was observed.

These findings suggest that plasma treatment improves cowpea degradability more effectively under higher salinity conditions, but excessive exposure can cause negative cellular changes. The optimal treatment is 1-minute exposure at low to moderate salinity levels (EC3.0 to EC5.5). Future research should optimize exposure duration and salinity conditions to maximize benefits.

#### Gas production parameters

 In vitro gas production is recognized as an indirect measure of ruminal fermentation efficiency and microbial activity, and therefore a reliable method of assessing nutrient usage^102^. In terms of salinity, there were no effects observed on GP/g DM and GP/g OM. Conversely, the GP/g NDF and GP/g ADF were significantly higher for plants grown at moderate salinity levels (EC5.5) and high salinity levels (EC7.0) compared to those grown at lower salinity levels (EC3.0). This suggests that salinity stress may alter fiber composition, promoting gas production per unit of fiber, even though there is no net change in total GP per DM.

Seed plasma treatment significantly affected in vitro gas production as GP/g DM and GP/g OM. Although plants grown from seeds exposed to plasma for only 1.0 min showed significantly higher INVDDM, INDNDF and INVDADF, the data showed that the same treatments significantly lowered GP/g DM and GP/g OM compared both the control seeds and the 2-minute treated seeds. This apparent discrepancy suggests a shift in fermentation pathways rather than a decrease in microbial activity. Gas production is primarily associated with the formation of CO₂ and CH₄, which are energetically inefficient end products for ruminal fermentation. Therefore, a reduction in gas production per gram of dry matter implies that a larger proportion of the fermented carbon may be redirected toward microbial biomass synthesis and/or the production of more reduced volatile fatty acids (VFAs), such as propionate. These VFAs generate less gas per unit of substrate fermented^103,104^. These findings align with studies that show treatments enhancing substrate availability can increase microbial growth efficiency, in which fermentable energy is preferentially incorporated into microbial protein rather than being lost as gaseous products^105,106^. Additionally, plasma-induced modifications of feed structure may alter fermentation kinetics, favoring propionate production over acetate. This change contributes to lower gas output while maintaining or improving digestibility^107^. In contrast, fiber-associated gas production (GP/g NDF and GP/g ADF) followed a different pattern. Plasma-treated plants exhibited enhanced fiber fermentation, particularly after 2-minute exposure, indicating improved fiber fermentation. This suggests that extended plasma treatment may modify the fiber matrix, improving its accessibility to microbial degradation. This hypothesis is further supported by the observed numerical increase (*P* = 0.0884) in volatile fatty acid (VFA) concentrations compared control treatment, which typically correlate with heightened microbial activity^32^.

The interaction between plasma exposure and salinity levels significantly affected the GP/g NDF and GP/g ADF measurements. This indicates that the impact of plasma treatment on fermentation dynamics is influenced by the salinity level. At EC 7, a 1-minute plasma treatment resulted in a reduction of GP/g ADF to 425.36 ml/g, compared to the control measurement of 472.51 ml/g. This suggests that at high salinity, the inhibitory effects of plasma may be alleviated. The 2-minute treatment yielded an intermediate value of GP/g ADF at 437.86 ml/g, indicating a more moderate fermentation response to the plasma treatment. In contrast, there were no significant interactions observed for GP/g DM and GP/g OM measurements, confirming that the effects of plasma treatment and salinity-related factors were mostly independent of each other.

#### Fermentation parameters

The fermentation parameters, including pH, ammonia (NH₃), and total volatile fatty acids (VFA), demonstrate the balance of microbial activity and nutrient availability. High salinity significantly increased ammonia levels (*P* ≤ 0.0001) compared to moderate and low salinity, which may be due to osmotic stress-induced accumulation of nitrogenous compounds or stress-related proteins, consequently increasing protein degradation and ammonia production. Plants under salt stress might absorb excess nitrogen compounds as osmolytes, which would be metabolized during the process of fermentation, releasing excess ammonia. This is consistence with findings of Khan et al.^83^, who observed similar perturbations of nitrogen metabolism caused by salinity.

The significant increase in ammonia concentration in plants grown from 2-minute plasma-treated seeds (*P* ≤ 0.0001) indicates that prolonged exposure to plasma increases proteolysis or microbial deamination during in vitro fermentation. This is likely due to the impact of plasmas on plant protein structures, which may allow microbial enzymes greater access to plant proteins. This suggestion aligns with Khan et al.^83^, who described changes in nitrogen metabolism pathways resulting from plasma exposure. Also, seed plasma treatment decreased antinutritional factors that impact protein degradation and consequently increased ammonia concentration. However, total VFA increases were non-significant across plasma treatments (*p* = 0.0857), Saudy et al.^32^ observed a significant 15% increase in TVFAs from 2-min plasma-treated seeds at EC7.0. This finding suggests that the duration of plasma treatment enhances microbial fermentation in saline conditions. Additionally, there was a numerical increase in VFAs in the plants treated for 2 min at E3.0 (9.28 meq/dl) was suggestive of improved fermentation efficiency in carbohydrate fermentation. This is consistent with Li et al.^88^, who showed that plasma stress increases the soluble carbohydrate available to the rumen microbiota, and as a result increase microbial VFA production. It should also be noted that plasma treatment did not affect pH (*p* = 0.3208), which reflects the ability of the plants to maintain constant fermentation acidity and buffering capacity over time.

The significant interaction between plasma treatment and salinity for ammonia (*p* ≤ 0.0001) and VFA (*p* = 0.0443) suggests context-dependent behavior. For instance, 2 min plasma treatment at EC3.0 gave the highest ammonia (11.91 mg/dl) and VFA (9.28 meq/dl), suggesting synergistic stimulation of microbial activity under low-salinity conditions. Conversely, at EC7.0, the same plasma treatment reduced VFA up to 7.83 meq/dl, suggesting that high salinity could counteract the benefits of plasma by limiting microbial access to fermentable substrates. The lowest ammonia content in 1-minute plasma-treated plants at EC5.5 (6.69 mg/dl) further demonstrates the movable balance between environmental stress and plasma dose.

Eventually, rumen degradation and fermentation characteristics are largely governed by forage chemical composition, substrate energy availability, and the presence of anti-nutritional compounds such as tannins and saponins. In the present study, the 1.0-minute plasma treatment resulted in higher organic matter (OM) content under normal conditions (low salinity), which likely contributed to its superior degradability and fermentation performance. Under moderate and high salinity, OM content became comparable between the 1.0- and 2.0-minute treatments, while the 1.0-minute treatment showed higher energy content, indicating that mild plasma exposure may better preserve readily fermentable substrates under saline stress. Crude protein content responded in a dose- and salinity-dependent manner, with the 0.1-minute treatment exhibiting higher CP under moderate and high salinity, whereas the 2.0-minute treatment showed higher CP under non-saline conditions. Moreover, the 1.0-minute treatment reduced saponin levels compared with both the control and the 2.0-minute treatment, while tannin concentrations were similarly reduced by both plasma durations relative to the control. Collectively, these results indicate that the 1.0-minute treatment provides the most balanced improvement in nutrient composition and anti-nutritional factor reduction across conditions, whereas the occasional advantages of longer plasma exposure at medium salinity likely reflect its greater ability to alleviate salinity-induced structural limitations. This pattern supports the existence of a salinity-modulated dose–response relationship, highlighting the need for future studies incorporating detailed fiber fractionation, fermentation end-product profiling, and microbial protein synthesis measurements to clarify the underlying mechanisms.

## Conclusions

This study demonstrates that cold plasma seed treatment effectively enhances cowpea’s tolerance to saline soils while improving its nutritional quality of cowpea for livestock feed. A 1.0-minute plasma exposure significantly reduced antinutritional factors (tannins and saponins) across varying salinity levels, enhancing fodder safety for ruminant consumption. Notably, plasma treatment synergized with salinity to improve digestibility. Furthermore, plasma treatment optimized ruminal fermentation by reducing fiber-associated gas emissions and boosting volatile fatty acid production. For practical applications, a 1.0-minute plasma treatment is recommended for maximal antinutritional factor reduction, whereas a 2.0-minute exposure enhances yield under moderate salinity (EC5.5). These findings underscore cold plasma as a promising tool for sustainable forage production in saline-affected regions.

Although the cowpea forage yield attributes were improved using NTP-treated seeds under normal or mild salt stress conditions, further investigations are needed to explore the physiological and molecular changes in the adult plant beside the seedlings.

## Data Availability

The datasets used and/or analyzed during the present investigation available from the corresponding author on reasonable request.
